# Fetal Programming of the Neuroendocrine-Immune System and Metabolic Disease

**DOI:** 10.1155/2012/792934

**Published:** 2012-08-16

**Authors:** R. E. Fisher, M. Steele, N. A. Karrow

**Affiliations:** Department of Animal and Poultry Science, University of Guelph, 50 Stone Road East, Guelph, ON, Canada N1G 2W1

## Abstract

Adverse uterine environments experienced during fetal development can alter the projected growth pattern of various organs and systems of the body, leaving the offspring at an increased risk of metabolic disease. The thrifty phenotype hypothesis has been demonstrated as an alteration to the growth trajectory to improve the survival and reproductive fitness of the individual. However, when the intrauterine environment does not match the extrauterine environment problems can arise. With the increase in metabolic diseases in both Westernized and developing countries, it is becoming apparent that there is an environmental disconnect with the extrauterine environment. Therefore, the focus of this paper will be to explore the effects of maternal malnutrition on the offspring's susceptibility to metabolic disorders such as obesity, cardiovascular disease, and diabetes with emphasis on programming of the neuroendocrine-immune system.

## 1. Introduction

Early life events such as those experienced *in utero* have the ability to shape the phenotype of an individual in an effort to prepare the fetus for extrauterine life. This is typically referred to as developmental or fetal programming and suggests that adverse uterine environments can “permanently” alter the metabolic, endocrine, and immune function parameters of individuals well into adulthood. There appear to be critical windows during development in which the fetus is most sensitive to environmental cues, altering the projected plan of growth. For instance, maternal adversity experienced during gestation will convey signals to the fetus that the environment in which it is to live is less than optimal, altering the developmental programming of the various organs and systems in the body to better match life outside the uterus. As various tissues and systems in the body mature and differentiate at different rates during fetal development, there appear to be critical periods in development when they are most sensitive to this adversity. The hypothalamic-pituitary-adrenal axis (HPAA), for example, undergoes much growth and differentiation during early and late gestation in species such as humans, primates, and sheep, and these are the periods when it is most sensitive to developmental programming alterations [1–3]. Alterations in metabolic function such as glucose tolerance and insulin sensitivity are greatly affected during mid- and late gestation as metabolic parameters are undergoing much differentiation during this period [4].

The alterations in developmental programming trajectories are assumed to provide an adaptive advantage for the individual to its new environment, referred to as the “thrifty phenotype”. A reduction in birth weight has been one alteration observed following adverse changes in the uterine environment. It is postulated that this alteration in growth pattern allowed the fetus to reallocate the available energy to more vital processes such as organ function [5]. The Leningrad wartime famine cohort of 1941 to 1944 is a classic example of the thrifty phenotype, demonstrating that maternal malnutrition during gestation primed the fetus by repartitioning metabolic energy for life in a malnourished society, ensuring survival and reproductive fitness [6]. However, when the developmental programming does not match the environment in which the individuals are to live, problems can arise.

## 2. Environmental Mismatch

Adverse uterine environments can occur for a variety of reasons such as maternal under- or overnutrition, maternal illness, or psychological stress that occurs during the critical windows in development. The amount of mismatch between the intrauterine and extrauterine environment can predict the individual's susceptibility to disease later in life [7]. Barker and colleagues were among the first to discover this association, coining the “Developmental Origins of Health and Disease” (DOHaD) hypothesis. They predicted that babies born with the lowest birth weight had the greatest risk of metabolic disease later in life, indicated by increased incidences of hypertension, obesity, and insulin resistance [8–10]. This hypothesis implies that adverse events that occur during fetal and neonatal development can increase susceptibility to adulthood diseases such as metabolic disease, although additional associations are being discovered. However, it should also be noted that the postnatal environment such as lifestyle (diet and exercise) as well as genetics play a large role in programming the offspring's susceptibility to disease; but will not be the focus of this paper.

The Dutch winter famine of 1944-1945 is a classic example of the DOHaD hypothesis. By using records from individuals whose mothers experienced the Dutch winter famine, researchers were able to make associations between individuals who experienced the famine during early gestation and increased susceptibility to hypertension, while those that experienced the famine during late gestation had an increased risk of impaired glucose tolerance and obesity during adulthood [4, 11]. These results emphasized how insults at various time points during gestation can differentially impact the various organs and systems of the body. Although the insult during gestation was the same as the Leningrad cohort, those that experienced the Dutch winter famine are a classic example of an environmental mismatch, as they were born into an environment in which nutrition had become abundant following the termination of war and therefore their metabolic “reprogramming” was deemed unnecessary for the environment in which they were to live.

An environmental mismatch is also becoming apparent in countries such as India as Westernized diets infiltrate the country. Typically, Indian babies are born small, ranging in birth weight between 2.6 and 2.7 kgs, and are thought to have an adaptive thrifty phenotype that is matched to their scarce food environment [12–14]. However, India is currently facing an epidemic crisis of adulthood type II diabetes and coronary heart disease (CHD) that is being linked to mismatched fetal and neonatal metabolic programming [15]. Indian children as young as 8 years of age, for example, have increased adiposity and insulin resistance putting them at increased risk of adulthood CHD [12].

Interestingly, this phenotype is also observed in individuals born to obese mothers. The rise in obese individuals in the population and the speculation that 68% of the US adult population has been reported to be obese has sparked interest into its effect on the programming of the fetus [16, 17]. Therefore, it is not surprising that offspring from obese mothers are more susceptible to childhood obesity, hypertension, insulin resistance, and type II diabetes [18–21].

Metabolic diseases have recently been classified as inflammatory disorders because they are accompanied by elevated concentrations of proinflammatory cytokines such as IL-1, IL-6, and TNF-*α*, as well as increased concentrations of glucocorticoids (GCs) in circulation [22–24]. Crosstalk between the innate immune system and the neuroendocrine system (i.e., HPAA) play an important role in regulating homeostasis in the body. For example, the excessive nutrients supplied to the hypothalamus in patients with metabolic disease have been implicated in causing inflammation and activation of the hypothalamus. These nutrients have been shown to induce oxidative stress, mitochondrial dysfunction, and stress in the endoplasmic reticulum triggering the activation of proinflammatory kinase pathways such as c-Jun N-terminal kinase (JNK) and inhibitor of B kinase (IKK). Excess nutrients have also been shown to activate Toll-like receptors (TLR) which also lead to the upregulation of JNK and IKK [25]. The increased concentrations of proinflammatory cytokines in individuals with metabolic disease may also act to stimulate the hypothalamus by crossing the blood-brain barrier leading to activation of the JNK and IKK pathways. Subsequent phosphorylation through JNK and IKK pathways leads to the induction of nuclear factors NF*κ*B and AP-1. NF*κ*B is an important regulator of inflammatory response genes leading to the activation of numerous proinflammatory cytokines, cytokine receptors as well as suppressor cytokine signaling 3 (SOC3), cyclooxgenase-2 (COX-2), and lipoxygenase [22]. Additionally, activation of the hypothalamus by these various mechanisms will also trigger the release of adrenal GCs. GCs play an important role in regulating the homeostasis in the body. GCs are released from the adrenal gland following activation of the HPAA. Briefly, during periods of perceived threats the HPAA becomes activated in order to achieve homeostasis. Once the body has recognized the threat it acts by secreting two neuropeptides corticotrophin releasing hormone (CRH) and arginine vasopressin (AVP) from the hypothalamus. These neuropeptides are then secreted into the hypophyseal portal to activate the pituitary for the production of adrenocorticotropin hormone (ACTH) from the anterior pituitary. Once produced ACTH can travel through the circulation to the adrenal cortex to activate the production of GCs. GCs play many important roles in the body from regulating fetal growth and development, mobilizing glucose and fat stores, controlling inflammation, and modulating the immune response [26–28]. Therefore, fetal programming-induced alterations in any aspect of these pathways have the ability to alter metabolic function leaving individuals susceptible to a variety of adult metabolic disorders.

Although metabolic diseases such as obesity, diabetes, and even cardiovascular disease have gained much attention in connection with the DOHaD hypothesis, various other diseases and disorders such as allergies, asthma, Alzheimer's disease, and psychological disorders are now also being linked with adverse uterine environments, although not covered in this paper. Therefore, the focus of this paper will be to examine the incidences and hypothesized mechanisms surrounding fetal programming of metabolic disorders.

## 3. Metabolic Disease

Metabolic disease encompasses a variety of different disorders, such as obesity, cardiovascular disease (CVD), and type II diabetes. With CVD and type II diabetes among the top ten leading causes of death in the United States, it is apparent that metabolic disease is quickly becoming an epidemic in the Westernized as well as the developing countries [29]. As metabolic diseases continue to climb within the world population, focus is being placed on their association with adverse uterine environments. Metabolic diseases were among the first recognized disorders associated with adverse uterine environments. Barker and colleagues first made this association by examining poverty stricken areas of England and Wales between 1968 and 1978, linking those individuals born with low birth weight to having an increased susceptibility to CHD as adults [30]. These associations were also confirmed through examination of individuals that lived through the Dutch famine of 1944-1945.

The Dutch famine provided a unique opportunity to examine the effect of undernutrition during different periods of gestation on the susceptibility to adulthood disease. Individuals born during the famine were typically lighter, shorter, and thinner than babies that were not exposed, representing an intrauterine growth restricted (IUGR) phenotype [31]. One of the concerns with IUGR individuals is that they may experience “catch-up” growth when exposed to an abundance of nutrition. Catch-up growth has been thought of as an adaptive survival mechanism, however, the long-term activation of both the growth and neuroendocrine systems increases the risk of metabolic diseases in adulthood [32]. For example, adults who were exposed to the famine during mid- or late gestation showed reduced glucose toleration, while those exposed during early gestation were shown to have a greater atherogenic lipid profile, higher body mass index (BMI), and a greater risk of CHD [33–36]. Type II diabetes was also more prevalent among the individuals who were exposed to the famine *in utero, *marked by reduced glucose tolerance and raised insulin concentrations among middle-age individuals [36, 37]. In support of this hypothesis recent work has demonstrated that children who were born small for gestational age (SGA) and experienced catch-up growth without obesity had higher rates of insulin resistance than children born appropriate for gestational age, suggesting that it is the uterine programming itself that influences the future outcome of disease [38]. Therefore, these studies suggest that alterations in uterine environment can influence the individual's susceptibility to metabolic disease on into adulthood; this will be more closely examined below.

## 4. Obesity

Ironically, both maternal under- and overnutrition has been shown to result in a similar obese offspring phenotype. This is becoming more and more apparent in developing countries such as India [39]. It has been discovered that although these children are born with the lowest birth weight, they have the smallest abdominal circumference, and a normal head circumference, they also surprisingly have the thickest skin-fold measurements suggesting that these babies are thin but with the greatest distribution of fat [40]. Work from Yajnik and colleagues [40] has shown that Indian babies tend to deposit fat tissue during development but have relatively poor tissue growth and protein turnover. What is of greatest concern, however, is that these children appear to retain this adiposity throughout childhood and into adulthood which could increase their susceptibility to other metabolic disorders [41].

Over the past few years overnutrition in both Westernized societies and developing countries has also become a concern as it is altering the metabolic programming of individuals making them more susceptible to obesity. With approximately two-thirds of American women being overweight, research is desperately needed in order to understand and prevent detrimental programming of the fetus [42]. Typically, offspring of obese mothers are born large and tend to have increased adiposity, making them further susceptible to metabolic diseases [43]. It has been speculated that maternal transfer of lipids is altered during an obesogenic pregnancy, often resulting in an earlier increase in lipid transfer compared to that of the pregnancy of a lean woman [44]. Briefly, in normal pregnancies, lipogenesis is greatest during early gestation in order to accumulate the fat stores required for a successful pregnancy [45]. Since obese mothers do not need to accumulate as many fat stores as leaner mothers, the process of lipid mobilization occurs much earlier in gestation and this means that more lipids can reach the fetus and this affects the programming of many organs and systems in the body associated with metabolism [44]. Additionally, the pregnancy of obese women has been shown to increase the hydrolysis of triglycerides and the concentration of free fatty acids as well as their transport across the placenta, once again leading to an increase in lipid exposure to the fetus [44]. Not surprisingly, the placentas from obese mothers also tend to support a proinflammatory environment as exhibited by increased expression of proinflammatory cytokines IL-1, TNF-*α*, and IL-6, as well as an increase in the accumulation of macrophages [46]. A recent study by Hayes and colleagues, [47], also demonstrated that an unfavourable uterine environment in rat dams fed a high-fat diet during pregnancy altered the placental vasculature which led to increased hypoxia during gestation, affecting not only the health of the offspring but also their viability. Under normal circumstances pregnancy is considered an inflammatory event associated with proinflammatory cytokines infiltrating the placenta. However, these cytokines are further increased in the obese mothers potentially due to the increase in both leptin and insulin concentrations [48]. Additionally, obese woman have an increase in the expression of CD14^+^ and CD68^+^ cells; two markers that define macrophages, and support the observed increase in proinflammatory cytokines [48]. The mechanisms behind placental lipid transport in obese pregnancies are generally understood; however, the mechanisms behind how it influences the fetal programming of metabolic states are generally not known.

The hypothalamus appears to be quite sensitive to nutritional programming as demonstrated through various animal models. This is not surprising as the hypothalamus houses the arcuate nucleus that contains the neurons for stimulating and suppressing appetite. Neurons containing neuropeptide Y (NPY) and GABA are found in the arcuate nucleus and are important in regulating appetite behaviour. Leptin and insulin, however, can inhibit the hormones of these neurons, thereby, suppressing an individual's appetite. Therefore, alterations in hormones and their receptors of this region of the hypothalamus can have a profound influence on the appetite behaviour of the individual. Alterations in the functioning of the leptin receptors as well as its secretion have been associated with obesity, overeating, and other metabolic diseases. For example, maternal high-fat diets have been demonstrated to induce leptin resistance in the hypothalamus of rat offspring, which led to an increase in body-weight gain during adulthood [49]. Other studies have also demonstrated increased concentrations of leptin in circulation of rats born to calorie-restricted mothers leading to leptin resistances and alterations in their feeding behaviour [50]. Additionally, male offspring born to the calorie-restricted mothers tended to consume more calories and gain more weight than control offspring [50]. The authors suggest that these effects could be explained by the lack of leptin surge that is normally observed between 6–14 days of postnatal development that is responsible for developing and differentiating the appetite control pathways in the hypothalamus. Other studies also support these findings as the mRNA expression levels of the leptin receptor in the hypothalamus of female rats born to mothers fed a high fat diet have been shown to increase, which is thought to be one reason for the observed increase in leptin levels [51]. However, it should also be noted that obese mothers or mothers with gestational diabetes have a higher concentrations of leptin in circulation that can reach the fetus affecting their programming [52].

The high level of triglycerides has also been proposed to play a role in altering the leptin profile of the fetus and increasing their susceptibility to adulthood disease. Studies have found that offspring leptin levels dramatically decline following parturition, possibly due to the higher concentration of maternal triglycerides in circulation [52]. Triglycerides have been shown to limit the amount of leptin crossing the fetal blood-brain barrier, creating somewhat of a leptin deficiency in these offspring [52]. This is concerning as studies have found that offspring with low postnatal leptin levels also tend to deposit fat tissue faster and gain more weight during lactation compared to their control counterparts [53].

Other studies have also reported changes in the STAT-3 pathway affecting the production of leptin [54]. The effects of leptin on other appetite controlling proteins such as neuropeptide Y are controlled through STAT-3. Briefly, once leptin is released from the adipose cell, it travels through circulation across the blood-brain barrier to bind to the leptin receptor in the hypothalamus. The intracellular domain of the leptin receptor can then bind to the STAT-3 resulting in its phosphorylation and activation of SOCS3 [55]. SOCS3 becomes an important regulator of the pathway, as it is capable of working through a negative feedback loop to downregulate the expression of leptin. Alterations in the STAT-3 pathway have been implicated in leptin resistance, which is commonly observed in obese individuals. Researchers have found significant decreases in the protein level of STAT-3 in the female rats from obese mothers, regardless of an increase in mRNA expression of STAT-3 and therefore alterations in this signal transduction pathway may affect appetite control [51].

Glucocorticoids have also been shown to be involved in fetal programming caused by maternal malnutrition. Higher levels of maternal GCs, for example, have been observed following gestational malnutrition, resulting in an increased concentration of GCs reaching the fetus and altering the HPAA programming [56, 57]. In a recent study by Belkacemi and colleagues [56], it was observed that undernourished rat dams had increased levels of plasma GCs in comparison to the control dams and this was accompanied by a reduction in fetal and placental weights. Furthermore, it was observed that there was an increased expression of 11beta-hydroxysteroid dehydrogenase (11beta-HSD) type 1 in the labyrinth zone of the placenta and a reduction in the expression of 11beta-HSD type 2, suggesting that there is an upregulation of active cortisol reaching the fetus [56]. An increase in GCs reaching the fetus has been associated with hypoleptinaemia and therefore may be one mechanism by which the hypothalamus is being reprogrammed [58, 59]. Interestingly, high-fat diets coupled with maternal stress appear to further exacerbate weight gain in rat offspring [60]. Offspring demonstrated an increased adiposity and a reduction in lean mass when their mother was fed a high-fat diet and stressed during gestation compared to offspring born to mothers fed the high-fat diet alone [60]. This trend continued following weaning with offspring experiencing a greater amount of weight gain compared to the control offspring. This is not surprising as GCs have been shown to increase the synthesis of endocannabinoids and their receptors, resulting in an increased appetite [61]. On the other hand leptin has been shown to block the endocannabinoid receptor downregulating appetite. Therefore, it appears that GCs and a high-fat diet may be working synergistically preventing leptin inhibition of this receptor. Overall, it is apparent that maternal under- and overnutrition play a large role in the development of the obesogenic phenotype. What is most concerning is that the obese phenotype observed in the offspring not only affects their overall quality of life but also increases their susceptibility to other metabolic disorders such as cardiovascular disease (CVD) and diabetes.

## 5. Cardiovascular Disease (CVD)

Cardiovascular disease was one of the first recognized disorders associated with adverse uterine environments. Fetally programmed individuals are more susceptible to hypertension and CHD and develop these disorders at much younger ages. For example, it was demonstrated using a rat model that offspring born to protein-restricted mothers had elevated systolic and diastolic blood pressures as early as four weeks of age, suggesting early development of CVD [62].

An increase in the occurrence of hypertension is commonly observed in offspring that were subjected to malnutrition during gestation. Individuals who were small for gestational age (SGA) at birth have been shown to have increases in blood pressure throughout childhood and adulthood, a trend that is interestingly not carried forward throughout adolescence [63]. In a recent study by Khoury and colleagues [64], it was demonstrated that waist-to-height ratios need to be considered when examining the susceptibility to hypertension and metabolic disease, as those individuals with increased weight-to-height ratios had greater adiposity and were more at risk of developing hypertension. It has been suggested that increased elasticity in vessel walls as well as alterations in placental enzyme 11beta-hydroxysteroid dehydrogenase type 2 (11beta-HSD2) may also be behind the increase in the susceptibility to hypertension [63, 65].

Glucorticoids as mentioned earlier, are typically elevated in mothers exposed to some degree of malnutrition during gestation, which increase fetal exposure to GCs [66, 67]. GCs play an important role in regulating cardiovascular function creating an environment that favours tissue sensitivity to vasoactive hormones [68]. GC actions are regulated by the enzyme 11beta-HSD, either promoting the active form of cortisol through enzymatic actions of 11beta-HSD1 or rendering cortisol inactive through 11beta-HSD2. The enzyme 11beta-HSD2 is an important regulator during pregnancy and is found in abundance at the level of the placenta where it protects the fetus from an overexposure to maternal GCs by rendering them inactive before they have the chance to cross the placental barrier. However, studies examining the effect of malnutrition on the development of offspring have demonstrated an inhibition in the activity of 11beta-HSD2 at the level of the placenta [67, 69]. In fact rat offspring born to mothers subjected to low-protein diets during gestation often exhibit an increase in glucocorticoid receptor (GR) expression and an attenuation in 11beta-HSD2 expression in various tissues of the body, such as the kidney, liver, and lungs which can be upwards of a two-fold increase compared to the control offspring [65]. Another research group has shown that dexamethasone administration to pregnant ewes in early pregnancy leads to reprogramming of the HPAA in the offspring, making them susceptible to hypertension approximately 5 years after the point of insult [70]. The impairment in both GR and 11beta-HSD2 in malnutrition-programmed individuals have the potential to influence sodium uptake, fluid-electrolyte homeostasis, and vascular tone; all of which have the potential to promote a favourable environment for hypertension in adulthood [65, 71, 72].

Subsequent studies have also demonstrated alterations in the renal-angiotensin system of offspring born to malnourished mothers. The renin-angiotensin system is an important regulator of cardiac function more specifically blood vessel constriction. Briefly, renin is a catalytic enzyme overseeing the conversion of angiotensinogen to angiotensin I, and subsequently angiotensin II. Angiotensin II is a potent vasoconstrictor leading to an increase in blood pressure, as well as the stimulation of aldosterone from the adrenal gland, which acts to increase the reabsorption of sodium and water. Maternal malnutrition has been shown to cause alterations in the gene and protein expression in many of the components of this system. For example, there is a decrease in both angiotensin I and II receptors in the kidneys of offspring born to low-protein-supplemented mothers [73]. When rat pups experienced IUGR due to maternal caloric restriction during gestation there was a significant upregulation in the angiotensin II in the kidney as well as a reduction in kidney weight [74, 75]. Therefore, it is not surprising that there are also increases in the angiotensin-converting-enzyme (ACE) and angiotensin-converting-enzyme 2 (ACE2) in rat offspring from undernourished mothers [75]. The increase in expression in both the enzyme and hormones responsible for vasoactivity nurture an environment supporting the grounds for hypertension. Alterations in the genes involved in sodium regulation such as renal Na/K-ATPase-alpha1 have also been observed to be affected by maternal malnutrition in the rat model; supporting the observation of elevated levels of renal Na/K-ATPase-alpha1 expression, in the offspring rats born to dexamethasone challenged mothers [76]. Similar results have been observed in sheep demonstrating a reduction in nephron number as well as alterations in the renin-angiotensin system of the brain [70]. Taken together these studies demonstrate associations between fetal programming of the renin-angiotensin system and the development of hypertension.

Placental insufficiency has also been shown to increase the susceptibility to adulthood CHD. In a cohort out of Helsinki Finland it was observed that individuals whose placenta measured less than 225 cm^2^ had an odds ratio of developing chronic heart failure if 1.7 compared to those individuals who had a normal placental distribution [77]. The odds were further increased in these individuals if the individual experienced rapid body-weight gain after the age of 2 and an increased body mass index at 11 years of age [77]. This seems to be further emphasized in the male population of the Helsinki cohort. When males were born SGA and remained small in infancy but had accelerated weight gain later in life, they had the greatest odds of developing CHD compared to the control men [78]. Similar results have been observed in a population of low and high birth weight boys. It was observed that boys who were born with a below-average ponderal index and rapid weight gain during childhood had a high rate of CHD among the study population; however, those boys who were born with an above-average ponderal index and experienced rapid weight gain during childhood did not show an association with CHD [79].

The increase in proinflammatory cytokines in obese individuals has also been shown to play a role in the development of CVD. For example, IL-1, IL-6, and TNF-*α* have been found to be increased in the circulation of obese people, which is not surprising as both have also been found to be produced in the adipose tissue [80]. Both cytokines promote an insulin resistant, high-triglyceride environment resulting in increased blood pressure and potentially CHD [81]. These cytokines have also been shown to increase the formation of plaque in the development of atherosclerosis as well as furthering the progression of the disease [82]. Furthermore, the amount of free fatty acids and TNF-*α* present in circulation of obese individuals increase the adhesion of monocytes to the endothelial monolayer which authors suggest could be influencing the inflammatory process and dictating the early development of artherosclerosis [83].

Lastly, fetal hypoxia has been shown to be associated with cardiovascular disease. As mentioned earlier, maternal obesity can lead to hypoxic conditions in the labyrinth of the placenta [47]. These hypoxic changes have the ability to alter the cardiovascular function in the offspring. For example, rats that were exposed to *in utero *hypoxic conditions demonstrated an increased susceptibility to ischaemia-reperfusion injury as adults [84]. Additionally, the cardiomyocytes of these rats were also enlarged with fewer numbers found in the heart [84]. In a recent study, increased likelihood of ischaemic injury caused by fetal hypoxia has been associated with an increase in the gene expression of angiotensin type II receptor (AT(2)R) in the rat [85]. Interestingly, there was a 50% decrease in the GR binding to glucocorticoid response elements in the AT(2)R promoter region in the hearts of these offspring [85]. Authors suggest that epigenetic mechanisms may be at play; however, the exact mechanisms are still unknown.

Overall, fetal programming appears to increase the susceptibility to cardiovascular disease. Although the exact mechanisms are unknown it is becoming clearer that both physiological mechanisms as well as alterations in genetic components are at play.

## 6. Type II Diabetes

Diabetes is also programmed by both over- and undernutrition and is currently reaching epidemic levels worldwide. Insulin sensitivity and resistance appears to be influential in the development of glucose tolerance and ultimately type II diabetes. Insulin sensitivity has been shown in many models of fetal origins of disease and appears to be a quite common consequence of malnutrition during gestation. Many of the studies of children who were born SGA have demonstrated a reduction in insulin sensitivity following a period of rapid catch-up growth during childhood. Insulin sensitivity in these children usually occurs in the presence of other factors such as increased adiposity and hypercholesterolemia [12, 86, 87]. Rapid catch-up growth appears to be one of the determining factors behind the development of insulin resistance in children born SGA. Children born in the Danish cohort study and who experienced rapid weight gain from birth to three months of age had higher insulin resistance at 1 year of age and higher basal insulin concentrations at 17.6 years of age, demonstrating that this alteration in metabolism is maintained [88]. Similarly, insulin concentrations were also observed to be higher in a cohort of healthy individuals born SGA compared to AGA [89]. Additionally, insulin resistance has also been shown to occur during fetal development in obese women compared to that of lean women suggesting that metabolic alterations are already apparent at the time of birth [90].

Various molecular mechanisms have been suggested for the increased susceptibility to insulin resistance and type II diabetes. For example, beta-cell development appears to be impaired following caloric restriction during pregnancy; this is exacerbated to the point of a permanent reduction in function if the mother is further restricted during lactation [91]. Similarly, rats whose mothers were fed a high-fat diet throughout gestation also demonstrated impairment in the beta-cell function, which was exacerbated in the female offspring compared to the males [92]. Epigenetic alterations have also been shown to be associated with reduction in beta-cell mass due to hypomethylation in the GR gene increasing the sensitivity to GCs [93]. Additionally, the promoter region of the PDX-1 gene, important for the normal differentiation of the beta cells, has been shown to undergo deacetylation at the histone complexes altering beta-cell development in IUGR rat pups [94]. Abnormalities in mitochondria may be to blame for these alterations in islet function. Mitochondria are imperative in the detection of glucose by beta cells and therefore alterations in their function can lead to impairments in this system [92, 95]. Gestational diabetes appears to further increase the susceptibility of type II diabetes in the offspring by targeting the beta cells [96]. Gestational diabetes also shows morphological impairment of the islets in the offspring, demonstrated through abnormal islet shape as well as hyperplasia and hypertrophy [97].

Islet function was also shown to be impaired in this rodent model [92]. Under normal circumstances islets increase the release of insulin upon an increase in the concentration of glucose found in the body. However, offspring born to both nutrient-restricted and high-fat supplemented mothers did not show an increase in insulin release following glucose stimulation indicating impairment in their function [92]. Glucose-stimulated insulin secretion was also impaired in islets from 9-month-old offspring born to dams that were fed a high-fat diet throughout gestation [98]. This study also revealed a reduction in expression of the mitochondrial genome in various tissues of the offspring.

Additionally, maternal obesity or high-fat diet during pregnancy has been associated with inflammation in the hypothalamus of the offspring, altering the TLR4 signaling cascade and increasing the offspring's susceptibility to diabetes. When TLR4 becomes activated following ligand binding, it subsequently activates both JNK and IKK pathways. Through multiple phosphorylation events in the IKK pathway NF*κ*B is produced from I*κ*B, which promotes the transcription of proinflammatory cytokines [99]. On the other hand phosphorylation of JNK will lead to the inhibition of insulin receptor substrate 1 (IRS-1) and ultimately insulin resistance in the host [100]. Therefore, these pathways play an important role in the development of insulin resistance and diabetes, and tend to be quite sensitive to fetal programming through maternal malnutrition. For example, a mouse study demonstrated that when dams were fed a high-fat diet from conception to weaning, their offspring had increased activation of JNK 1 and IkB kinase [101]. Additionally, tissue samples collected from rats fed a Westernized diet demonstrated an increase in insulin resistances in tissues that had the greatest JNK activity and IRS-1 phosphorylation [102]. Although the mechanisms by which alterations in the TLR4 signaling cascade can lead to insulin resistance and type II diabetes is not known, it is becoming increasingly apparent that it plays a role in the susceptibility. A mouse strain, CeH/HeJ, further supports this idea, as these mice have a mutation causing loss of function in TLR4 protecting them from diet-induced obesity and insulin resistance [103, 104].

Therefore, it appears that the intrauterine environment can increase the susceptibility of diabetes later in life due to alterations in insulin sensitivity and changes in beta-cell expression and mass.

## 7. Summary

It is becoming more apparent that adulthood metabolic disease is associated with adverse uterine environments. Although adversity is thought to prime the offspring for life outside the uterus the diverse phenotypes in the offspring from malnourished mothers is increasing their susceptibility to adulthood metabolic diseases such as obesity, CVD, and diabetes. With metabolic disorders reaching epidemic levels in the world population, future research is required in order to understand the underlying mechanisms leading to alterations in the programming of the fetus and subsequent development of adulthood disease. What is apparent is that the neuroendocrine-immune system interactions play a key role in the development of metabolic disease. With an increase in knowledge of these interactions future emphasis can be placed on the treatment and prevention of metabolic disorders of fetal origin.
